# Case report: An overlooked complication of the dural suture after craniotomy: pseudoaneurysm of the middle meningeal artery with endovascular resolution

**DOI:** 10.3389/fneur.2023.1173821

**Published:** 2023-05-05

**Authors:** Yao Xu, Xiangdong Li

**Affiliations:** Department of Neurosurgery, The First Affiliated Hospital of Soochow University, Suzhou, China

**Keywords:** aneurysm, cerebral angiography, case report, middle meningeal artery (MMA), craniofacial surgery

## Abstract

Aneurysms of the middle meningeal artery (MMA) are exceedingly uncommon and mainly result from traumatic brain damage, but this report describes a case of MMA aneurysm induced by cranial surgery. Surgery was performed on a 34-year-old male with cerebrovascular malformation and cerebral hemorrhage. Cerebral angiography revealed no MMA aneurysm before craniocerebral surgery; however, a postoperative angiogram revealed a new MMA aneurysm. Aneurysms of the MMA are uncommon consequences of brain surgery. Based on our findings, the MMA as well as other meningeal arteries should be avoided while suturing the dura mater tent to prevent aneurysms.

## Introduction

Aneurysms of the middle meningeal artery (MMA) are exceedingly rare. Rupture can result in a variety of intracranial hemorrhages, including epidural haematomas (1), subdural hematomas, and intracerebral hemorrhage (2).

True aneurysms and pseudoaneurysms are the two forms of aneurysms. True aneurysms of the MMA resemble regular cerebral aneurysms, typically originating from their branches, and are usually related to increasing haemodynamic stress or a pathological state in MMAs. Increased blood flow and haemodynamic stress can be caused by many disorders, including arteriovenous malformations (3), Moyamoya disease (4), meningioma (5), and cranial tumor metastases (6). In addition to haemodynamic stress, other pathological states of the MMA, such as Paget's disease, hypertension (7), and type 2 neurofibromatosis (8), can cause aneurysms.

The etiology of pseudoaneurysms of the MMA is either traumatic or medically induced injury. A temporal skull fracture generates a tiny rupture in the vascular wall, which is subsequently occluded by a clot during the acute phase before recanalisation to establish a false lumen. On angiography, pseudoaneurysms frequently lack a neck and are uneven, resulting in delayed and extremely sluggish filling and emptying (9), and possible iatrogenic damage.

In the present case, no obvious MMA aneurysms on angiogram were reported before surgery and prior to discharge. We present the case of a 34-year-old male with an MMA aneurysm which was discovered on an angiogram obtained 12 days after cranial surgery.

## Case report

**Figure 1:**
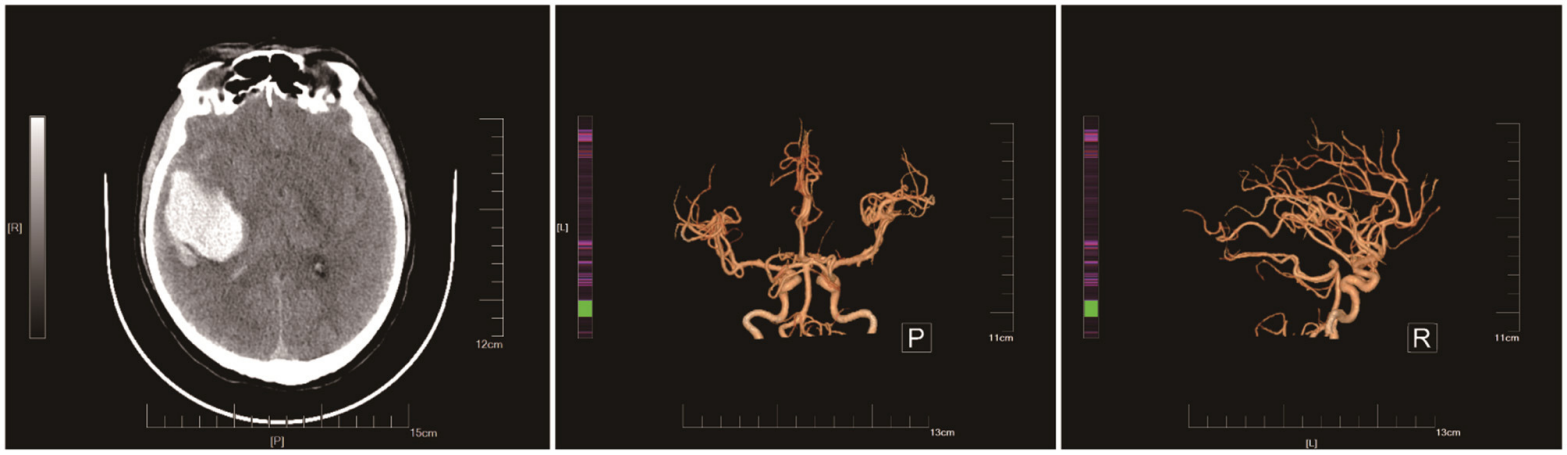
Preoperative brain CT images show a right temporal lobe cerebral hemorrhage and a diffuse subarachnoid hemorrhage, suggesting an Arteriovenous Malformation (AVM).

**Figure 2:**
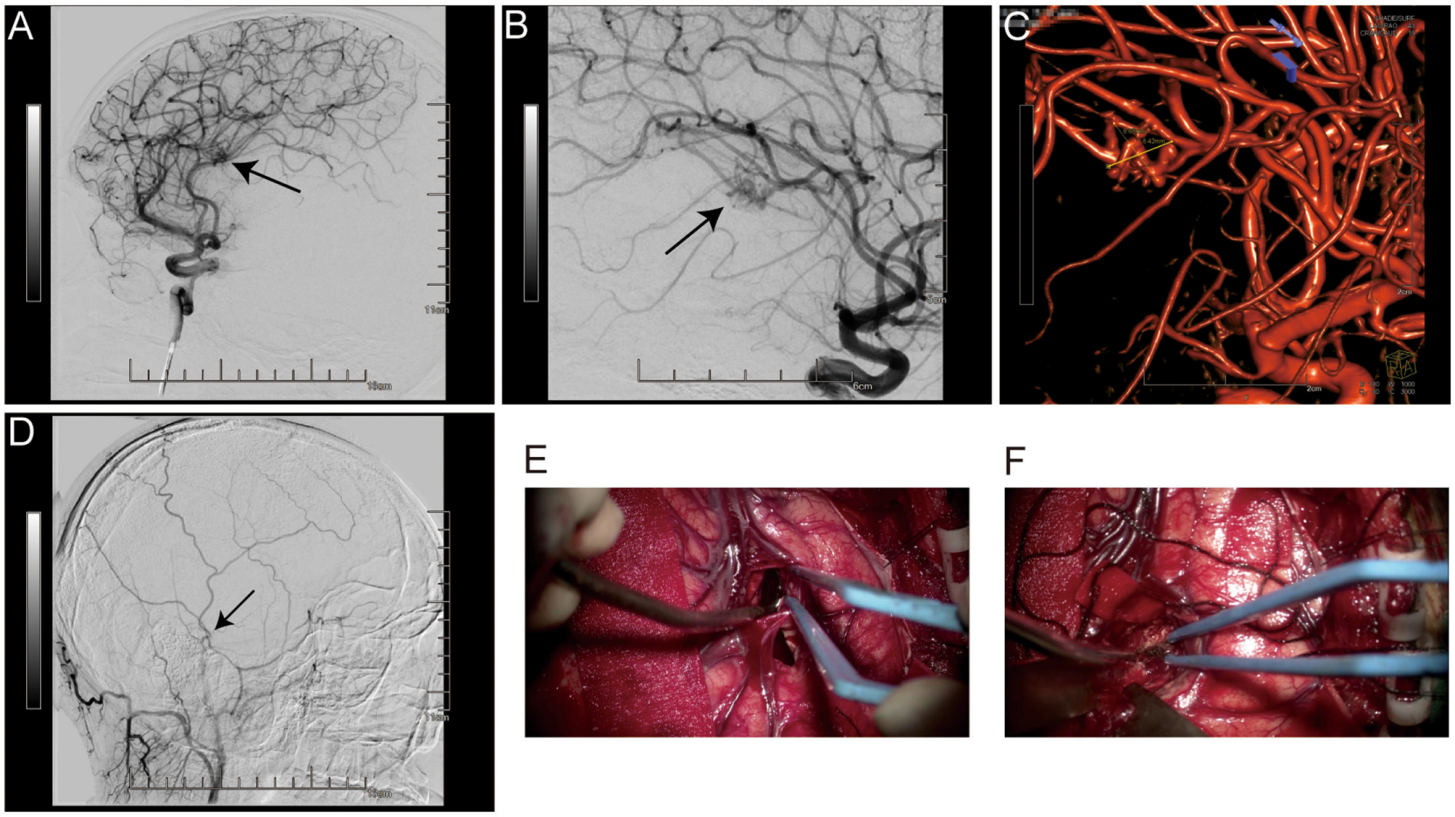
Before surgery, a right internal carotid cerebral angiography revealed an 8.9 × 7.4 mm vascular malformation in the right temporal lobe, which is fed by the ipsilateral middle cerebral artery's inferior trunk branch **(A–C)**. The MMA was free of aneurysms **(D)**. The surgical images show a blood clot and a distorted vascular mass in the insular cortex **(E, F)**.

A 34-year-old man presented with loss of consciousness that lasted for 5 h, accompanied with headache and vomiting; hence, he was admitted to the hospital. Computed tomography angiography (CTA) of the brain revealed cerebral hemorrhage in the right temporal lobe and diffuse subarachnoid hemorrhage, raising the possibility of an arteriovenous malformation (AVM) (Figure 1). Subsequently, the patient was transferred to our department for surgery. Intraoperatively, an 8.9 × 7.4 mm vascular malformation in the right temporal lobe, which was fed by the inferior trunk branch of the ipsilateral middle cerebral artery, was discovered on right internal carotid cerebral angiography (Figures 2A–C) (Supplementary Video S1). The Spetzler-Martin AVM grade is 2, and the supplementary grade is 2. No aneurysm was observed in the right MMA during this time (Figure 2D). The patient's family actively requested surgical treatment. Cerebral vascular malformation excision was performed. Surgical footage showed a blood clot and a distorted vascular mass in the insular cortex (Figures 2E, F). The M3 branch of the middle cerebral artery supplies the blood to the AVM. No drainage veins were seen on DSA, tiny drainage vein was seen during craniotomy. There were no evident dural lacerations or leakage from the MMA during the operation. The patient regained consciousness after surgery with minimal neurological sequelae, and a brain CT scan the next day revealed no cerebral infarction or apparent epidural or subdural hemorrhages.

**Figure 3:**
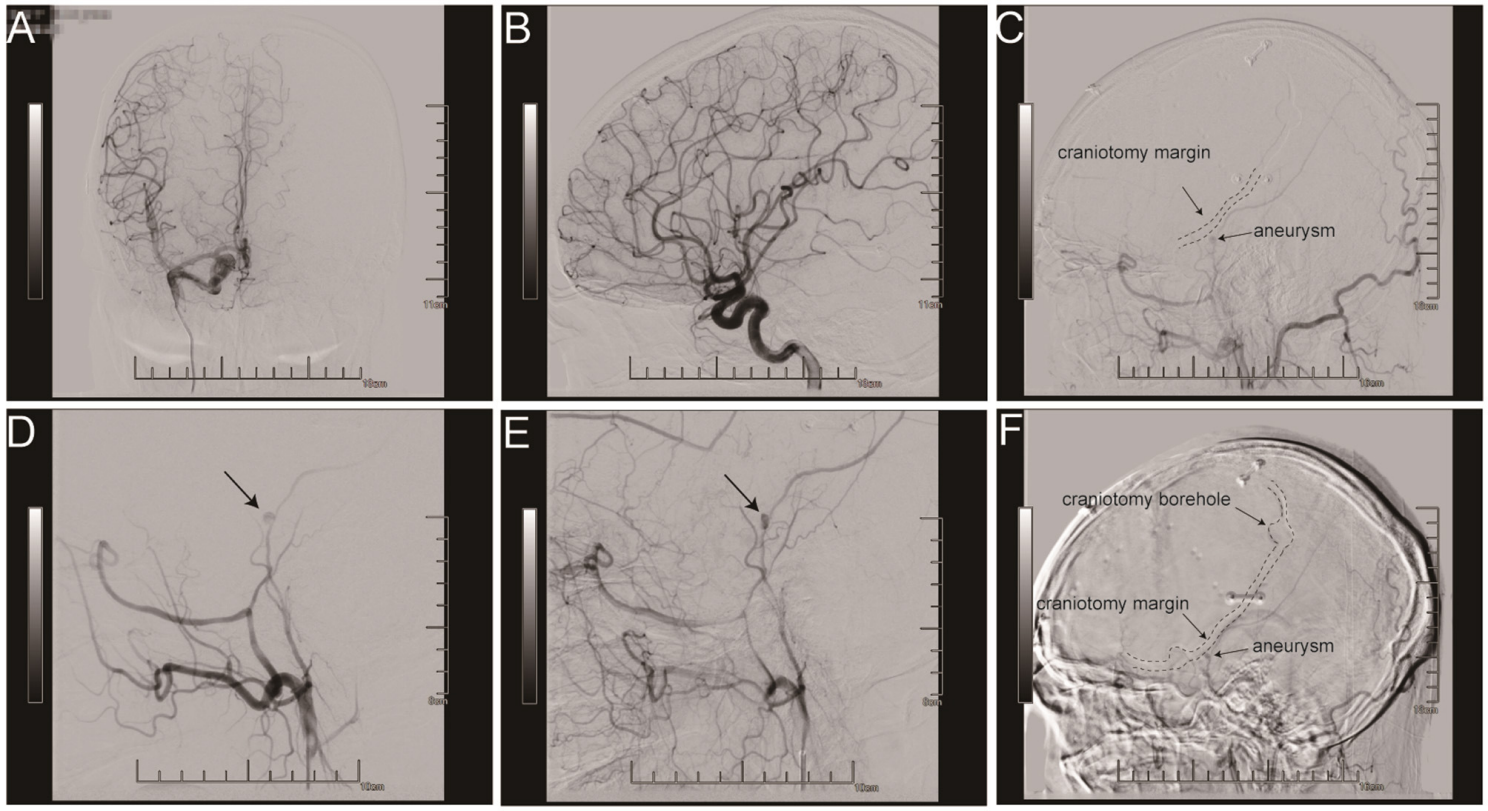
A full resection of a cerebral vascular abnormality was identified during the evaluation **(A, B)**. In the cerebral angiography following surgery, a 3.3 × 3.1 mm aneurysm of the right posterior MMA was discovered at the craniotomy cut edge of the skull **(C, F)**. Angiograms demonstrating late filling of a MMA aneurysm and contrast medium stagnation **(D, E)**.

**Figure 4:**
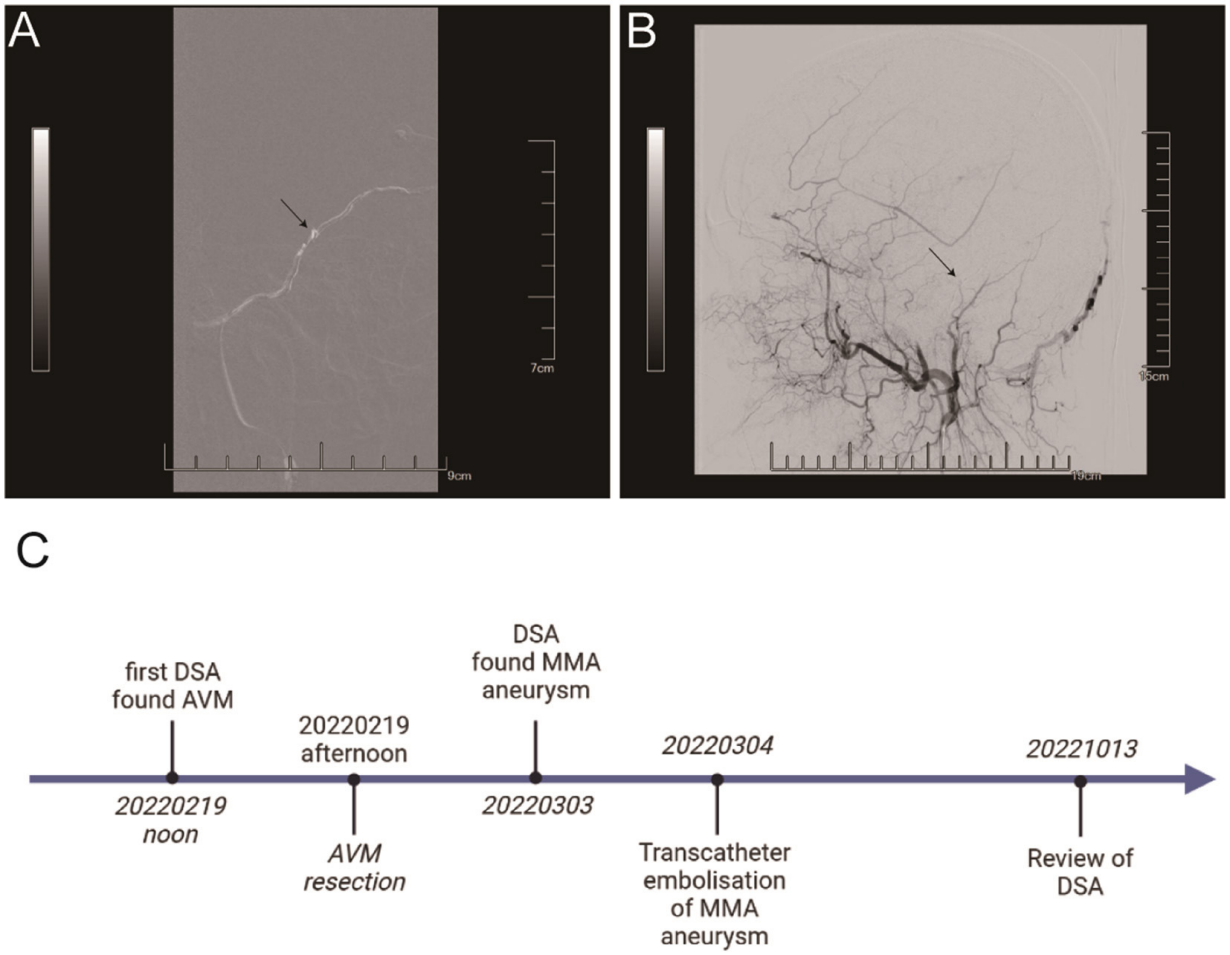
The ONYX 18 embolic agent was injected into the right MMA posterior branch **(A)**. There was no evidence of an aneurysm or the posterior branch of the right MMA **(B)**. It's the timeline of the patient receiving treatment and follow up **(C)**.

Cerebral angiography was conducted 12 days after surgery, which revealed that the cerebral vascular abnormality was completely removed (Figures 3A, B); however, a 3.3 × 3.1 mm posterior right MMA aneurysm (Figure 3C) (Supplementary Videos S2, S3) was seen, which was absent on initial cerebral angiography. Because angiography demonstrated late filling of the aneurysmal sac during the arterial phase and contrast medium stagnation, this MMA aneurysm was classified as a pseudoaneurysm (Figures 3D, E). The aneurysm was visible near the cut edge of the skull in the images (Figure 3F). We informed the patient and family that the aneurysm was at risk of rupture. They can choose between endovascular intervention, craniotomy and no further treatment. The patient and family opted for the less invasive endovascular treatment. Endovascular embolisation was performed with an ONYX 18 embolic agent. Right external carotid arteriography revealed the MMA aneurysm found on the last DSA. A 5 ml ONYX 18 embolic agent was progressively injected through a microcatheter into the posterior branch of the MMA (Figure 4). The patient was allowed out of bed and was able to ambulate 2 days after embolisation. The patient provided informed consent to have his case published.

The patient came to our hospital for follow-up after 6 months. DSA revealed no recurrence of AVM and MMA aneurysm (Supplementary Videos S4, S5). Here is the timeline of the patient receiving treatment and follow up (Figure 4C).

## Discussion

The pathogenesis of the MMA aneurysm in our case was directly linked to surgery, since it was not initially seen on preoperative angiography but was detected on postoperative angiography. Based on the digital subtraction angiography (DSA) images (Figure 3), we concluded that the MMA aneurysm was induced by dural tenting sutures and craniotomy. We carefully examined the edges of the dura mater during the operation. If the aneurysm was caused by craniotome, then intraoperative hemorrhage and a rupture of the dura mater will be found.

A previous study described an MMA pseudoaneurysm following internal carotid artery trapping and high-flow bypass with a radial artery graft (10). Another study presented an iatrogenic pseudoaneurysm of the MMA after installation of an external ventricular drain (11). However, these studies lacked comprehensive preoperative and postoperative imaging data. Iatrogenic MMA aneurysms were observed at the skull and dural borders in all three cases. According to our imaging data, MMA aneurysms were linked to craniocerebral surgery. These examples imply that dural tenting sutures may be associated with MMA aneurysms.

Observations made on a number of patients, including the case presented, reveal the occurrence of epidural and subdural hemorrhage following brain surgery. Since angiography was either not done or initially showed irrelevant findings, these cases have been linked to the placement of dural tenting sutures.

Most pseudoaneurysms and real aneurysms require treatment owing to the risk of rupture. Endovascular embolisation and surgical resection with haematoma evacuation when needed are the treatments of choice for these diseases.

## Conclusion

The MMA is a critical landmark when performing brain surgery. The position of the MMA and other meningeal arteries should be meticulously considered when suturing dural tents since it is prone to iatrogenic injury.

## Data availability statement

The original contributions presented in the study are included in the article/Supplementary material, further inquiries can be directed to the corresponding author.

## Ethics statement

Written informed consent was obtained from the individual(s) for the publication of any potentially identifiable images or data included in this article.

## Author contributions

YX wrote the manuscript. XL suggested ideas and provided guidance. Both authors contributed to the article and approved the submitted version.
